# Seasonal genetic variation associated with population dynamics of a poecilogonous polychaete worm

**DOI:** 10.1002/ece3.3518

**Published:** 2017-10-22

**Authors:** Anne Thonig, Gary Thomas Banta, Benni Winding Hansen, K. Emily Knott

**Affiliations:** ^1^ Department of Science and Environment Roskilde University Roskilde Denmark; ^2^ Department of Biological & Environmental Science University of Jyvaskyla University of Jyvaskyla Finland

**Keywords:** bet‐hedging, chaotic genetic patchiness, developmental mode, poecilogony, *Pygospio elegans*

## Abstract

Poecilogonous species show variation in developmental mode, with larvae that differ both morphologically and ecologically. The spionid polychaete *Pygospio elegans* shows variation in developmental mode not only between populations, but also seasonally within populations. We investigated the consequences of this developmental polymorphism on the spatial and seasonal genetic structure of *P. elegans* at four sites in the Danish Isefjord‐Roskilde‐Fjord estuary at six time points, from March 2014 until February 2015. We found genetic differentiation between our sampling sites as well as seasonal differentiation at two of the sites. The seasonal genetic shift correlated with the appearance of new size cohorts in the populations. Additionally, we found that the genetic composition of reproductive individuals did not always reflect the genetic composition of the entire sample, indicating that variance in reproductive success among individuals is a likely explanation for the patterns of chaotic genetic patchiness observed during this and previous studies. The heterogeneous, unpredictable character of the estuary might maintain poecilogony in *P. elegans* as a bet‐hedging strategy in the Isefjord‐Roskilde‐Fjord complex in comparison with other sites where *P. elegans* are expected to be fixed to a certain mode of development.

## 1 INTRODUCTION

Phenotypic variation within a single population or species is a classical focus of ecology and evolution, and both the causes and consequences of polymorphism are actively investigated (e.g., Schwander & Leimar, 2011; Wennersten & Forsman, 2012). Even though phenotypic variation can arise via different mechanisms, for example, genetic polymorphism, developmental plasticity or randomized switching, the consequences for populations can be very similar. Populations with high phenotypic variation are expected to have larger niche breadths and increased colonization potential, as well as decreased intraspecific competition, decreased vulnerability to environmental changes, and decreased fluctuations in population size (Wennersten & Forsman, 2012). However, the consequences of phenotypic variation are also influenced by what kind of traits show variation. For example, variation in life‐history traits is likely to have strong effects on colonization potential and fluctuations in population size, whereas traits affecting nutrient acquisition might have a stronger effect on intraspecific competition (Wennersten & Forsman, 2012).

Variation in developmental mode, when a single species produces different types of larvae, is called poecilogony. Poecilogony is known only in some marine invertebrates (notably among spionid polychaete worms and sacoglossan sea slugs), and the degree of variation in developmental mode can differ between poecilogonous species (see Collin, 2012; Knott & McHugh, 2012; McDonald, Collin, & Lesoway, 2014). For example, variation in developmental mode can occur between populations, between females within the same population, between broods of the same female, or even within broods. Likewise, there are different possible mechanisms allowing for poecilogony, including fixed genetic polymorphisms, plasticity in response to environmental cues, or maternal effects (Collin, 2012; Knott & McHugh, 2012). Because the larval stage of benthic marine invertebrates has a significant impact on their dispersal ability, variation in the mode of development has consequences for spatial and temporal population genetic structure (Collin, 2001; Cowen & Sponaugle, 2009; Eckert, 2003; Lee & Boulding, 2009). High population connectivity and low spatial genetic structure are expected for species with planktonic larvae due to their higher dispersal potential in comparison with species with non‐planktonic larvae (Bohonak, 1999; Hellberg, 2009). However, dispersal potential does not always translate into realized dispersal and connectivity and might not predict population genetic structure (e.g., Weersing & Toonen, 2009). Moreover, temporal fluctuations in genetic structure can occur in species with planktonic larvae due to sweepstakes reproductive success, particularly in highly fecund species, and/or due to selection during the planktonic phase (Hedgecock & Pudovkin, 2011; Lee & Boulding, 2009).

One species exhibiting poecilogony is *Pygospio elegans*, a small (max. 20 mm), tube‐dwelling spionid polychaete with an average life span of 9 months, which exhibits a broad range of habitat tolerances, population densities, and a variety of feeding modes (Anger, 1984; Anger, Anger, & Hagmeier, 1986; Hempel, 1957). It can reproduce asexually via fragmentation (Anger, 1984; Rasmussen, 1953), whereas embryos resulting from sexual reproduction are laid in egg capsules within the mother's sand tube. Larvae spend part of their development within the egg capsules feeding on unfertilized nurse eggs provided by the mother (oophagy). When there are few embryos (<4) and many nurse eggs, the larvae are classified as benthic larvae: These hatch from the egg capsules at a large size and do not have a large potential for dispersal. In contrast, when there are many embryos (>10) and few or no nurse eggs laid in the capsules, the larvae are classified planktonic larvae: These hatch at a small size and complete their development in the plankton and have a greater potential for dispersal. However, the difference between benthic and planktonic larvae is not always discrete, and intermediate larvae also exist (Rasmussen, 1973; Thonig, Knott, Kesäniemi, Winding Hansen, & Banta, 2016). The association of developmental mode and nurse egg production suggests a possible maternal effect (as noted for other poecilogonous polychaetes, e.g., Oyarzun & Brante, 2014). However, the underlying mechanism of poecilogony in *P. elegans* is still not known, and multiple mechanisms might work in concert. For example, different developmental modes in *P. elegans* are found both between populations and within populations, at times showing seasonal switches (Gudmundsson, 1985; Rasmussen, 1973; Thonig et al., 2016), suggesting a possible environmental influence. Also, the possibility of genetic polymorphism has been suggested because some populations are presumed to have a fixed developmental mode (Bolam, 2004; Morgan, Rogers, Paterson, Hawkins, & Sheader, 1999), and this possibility has not been ruled out.

In the first part of this study, we investigated the population dynamics of the poecilogonous spionid *P. elegans* at four sites in the Danish Isefjord‐Roskilde Fjord estuary and how environmental parameters might affect the population dynamics (Thonig et al., 2016). We identified two main recruitments, seen as the appearance of new size cohorts, one in spring and one in fall. Previous cohorts seemed to disappear during summer and winter, thus resulting in a turnover of the population. Sexual reproduction occurred predominantly from September until May. These results confirmed observations of Rasmussen (1973), Gudmundsson (1985), and Bolam (2004). Two separate peaks of gravid females were observed at three of four sites, and these showed a switch in type of larvae from planktonic larvae in winter to intermediate and benthic larvae in spring. One peak of gravid females and only intermediate and benthic larvae were observed at the innermost site, Herslev. The seasonal population dynamics were related to temperature, with reproduction occurring at low temperature. Median grain size and sorting of the sediment correlated with the spatial differences, where higher densities of *P. elegans* and larger specimens were observed at sites with coarse and poorly sorted sediment (Herslev and Vellerup).

In this part of the study, we analyzed the population genetic structure of *P. elegans* using seven microsatellite loci to genotype individuals sampled from the same four locations at six different time points over 1 year. Our aim was to determine whether genetic differences among individuals and cohorts are associated with the population dynamics we described in Thonig et al. (2016). Previous studies of population genetic structure in *P. elegans* have not been able to adequately follow individual worms with known developmental modes, but rather examined populations with different larval types, or populations categorized based on the developmental mode observed in a sample (e.g., Kesäniemi, Boström, & Knott, 2012; Kesäniemi, Geuverink, & Knott, 2012). Here, we compare genotypes and phenotypes of individuals sampled both spatially and temporally to describe consequences of poecilogony on the population genetic structure of *P. elegans*.

## 2 MATERIALS AND METHODS

### 2.1 Sampling

We conducted a field survey from March 2014 until February 2015 to document the population dynamics of *P. elegans* at four sampling sites in the Danish Isefjord‐Roskilde‐Fjord estuary complex: Lynæs, Lammefjord, Vellerup, and Herslev (described in detail in Thonig et al., 2016). In this study, we examine population genetic structure at the four sites from samples collected at six time points (in March, May, August, October, November, and February) in order to determine whether genetic differences can be detected between size cohorts and how variation in developmental mode is related to the spatial and temporal population genetic structure. *Pygospio elegans* were sampled from the top layer of sediment and sieved on site with a 1‐mm mesh. In the laboratory, subsamples of 27–44 individuals were sized (Thonig et al., 2016) and afterward stored in 99% ethanol for DNA extraction.

### 2.2 DNA extraction and microsatellite genotyping

DNA was extracted from whole individuals using the Qiagen DNeasy Blood & Tissue Kit following the manufacturer's protocol for animal tissue. We developed two multiplex reactions to amplify ten microsatellite loci in *P. elegans*. Seven of the microsatellite loci were identified from a draft transcriptome of *P. elegans* (Heikkinen, Kesäniemi, & Knott, 2017), and primers were designed to amplify these loci using WebSat software (Martins, Lucas, Neves, & Bertioli, 2009). Three of the loci (Pe6, Pe7, and Pe19) were described previously (Kesäniemi, Boström et al., 2012) (see Table 1). Multiplex PCR reactions of 10 μl were performed containing 1x Qiagen Multiplex PCR Master Mix, 0.2 μmol/L of each primer, and 1 μl DNA template (diluted 1:20). The PCR had an initial activation step of 15 min at 95°C followed by 30 cycles of 30 s at 94°C, 90 s at 60°C, and 60 s at 72°C, and a final extension for 30 min at 60°C. Fragments were separated using an ABI PRISM 3130xl Genetic analyzer with Gene Scan™ 500 LIZ™ size standard (Applied Biosystems) in our own laboratory. The results were analyzed with GeneMapper^®^ v.5 Software (Applied Biosystems).

**Table 1:** Microsatellite loci, repeat found in reference sequence, the primers used for amplification, and GenBank accession number

Locus name	Repeat sequence	Primer sequences	GenBank accession number	No. of alleles	Size range (bp)
Pe307	(TG)6	F: AGCTAAATCTTGACACTGGCCT	MG021816	12	181–202
		R: GAAGTCAGCCATCTTGGATTCT			
Pe309*	(ATG)8	F: CCAGAGGAAATGATGTAGGCTC	MG021817	11	377–402
		R: ATTCACACTTGACCATGACCAC			
Pe385	(GGT)8	F: TCAATAGGAGAAGCACAACGAA	MG021818	13	392–430
		R: CGCTGGTTATTTTAGGGATGAG			
Pe6	(CA)28	F: ACTACGGAAACTGCCTGCAC	GU321899	6	265–287
		R: ATATGGCCACCGAAACCTCT			
Pe7*	(CATA)13	F: CTCACCCTTTACACCCAAGG	GU321900	38	124–255
		R: AGCGTCTGTTATGGGGTACAG			
Pe19	(GA)23	F: TATCCAACGCACACCTACCA	GU321906	13	214–285
		R: TTGAGTGATGGTGCGAGGTA			
Pe159*	(GT)10	F: TTGGTTTGAGCAATGTGGAA	MG021819	35	184–255
		R: GCCCTTTGCACTCATTGTTT			
Pe234	(AG)6AA(AG)4	F: AGCAGTAAAAGCGGATCACAAC	MG021820	5	374–384
		R: TGTCTCTGGCGTAATTTTCTCA			
Pe294	(AG)5	F: AGTGGGTGTGTGAGAAGAGC	MG021821	5	231–239
		R: AGTTGAGCCGTGATACAAAATC			
Pe369	(GT)8	F: CTTTCTTCCCCAAGGCTTCT	MG021822	17	190–227
		R: TTTCTCACCCTCCTGACCTG			

Loci marked with an asterisk were discarded from the study because they showed a high estimated null allele frequency. The number of alleles and size range observed in this study are shown. Loci Pe6, Pe7, and Pe19 were described in Kesäniemi, Boström et al. (2012). The loci were grouped into two multiplex panels: Multiplex 1 contained Loci Pe307, Pe309, Pe385, Pe6, and Pe7; Multiplex 2 contained Loci Pe19, Pe159, Pe234, Pe294, and Pe369.

### 2.3 Quality of loci

To ensure the quality of the data, every allele that occurred only once in the data set was double‐checked and confirmed in the raw data. Individuals missing information for more than two loci were discarded. Three loci had more than 5% missing data (Pe7—5.7%; Pe159—6.1%; and Pe309—8.1%) and were suspected to have null alleles. We used Micro‐Checker (Van Oosterhout, Hutchinson, Wills, & Shipley, 2004) to estimate null allele frequencies for all loci and found that loci Pe7, Pe159, and Pe309 had a significant proportion of null alleles (Oosterhout calculation: up to 2.2% in Pe7, 2.9% in Pe309, and 3.3% in Pe159) in many of the samples. Locus Pe385 also showed possible null alleles, but these were always less than 2%, which is not expected to affect downstream analyses significantly (Putman & Carbone, 2014). Gametic disequilibrium and Hardy–Weinberg equilibrium (HWE) were checked per locus and sample using Fstat v.2.9.3.2 (Goudet, 1995). Loci Pe7 and Pe159 were not in HWE in the majority of the samples. Consequently, we decided to eliminate three loci from the data set: Pe7, Pe159, and Pe309. Therefore, further statistics are calculated based on data from the remaining seven polymorphic loci (see Table 1). An outlier test was performed in LOSITAN (Antao, Lopes, Lopes, Beja‐Pereira, & Luikart, 2008; Beaumont & Nichols, 1996) for all loci except Pe7 and Pe159 using “Neutral mean *F*_ST_,”, “Force mean *F*_ST_,”, and “100,000” simulations. This test indicated that Pe385 might be subject to positive selection and Pe294 might be subject to balancing selection.

### 2.4 Genetic diversity

For each sample, observed and expected heterozygosity (*H*_o_ and *H*_e_), gene diversity, and *F*_IS_ averaged overall loci were calculated using Arlequin v.3.5.2 (Excoffier & Lischer, 2010). *F*_IS_ was calculated for each sample separately, assuming no temporal or spatial groups, from a distance matrix based on the number of different alleles, and 20,000 permutations were performed to calculate the *p*‐values. We calculated allelic richness and number of private alleles using the rarefaction method implemented in HP‐Rare v1.1 (Kalinowski, 2005). These values are calculated based on the same number of individuals per sample to enable comparisons between samples. Relatedness within each sample was calculated using the triadic likelihood estimator implemented in Coancestry v.1 (Wang, 2007, 2011), which infers allele frequencies from the genotypic data and accounts for inbreeding. Hereby, 100 individuals are used as a reference sample, and 100 bootstrapping samples were used to calculate the 95% confidence intervals.

### 2.5 Population structure

Population structure was analyzed using three different approaches. Firstly, analysis of molecular variance (AMOVA) was performed in Arlequin v.3.5.2 (Excoffier & Lischer, 2010). For AMOVA, samples were grouped in either temporal or spatial groups. The distance matrix used in the analysis was based on the number of different alleles, and the *p*‐values were calculated based on 20,000 permutations. Secondly, population differentiation was estimated using $G_{\mathrm{ST}}^{\prime }$ (Hedrick, 2005) and Jost′s D (Jost, 2008) statistics implemented in the R package diveRsity (Keenan, McGinnity, Cross, Crozier, & Prodöhl, 2013) for each pair of samples. The correlation between different statistics (*F*_ST_, *G*_ST_, $G_{\mathrm{ST}}^{\prime }$, and Jost′s D) and the mean number of alleles for each locus showed a similar trend (data not shown). Thirdly, the model‐based clustering method implemented in Structure v.2.3.4 (Pritchard, Stephens, & Donnelly, 2000) was used to assign individuals to distinct clusters. We used the admixture model, correlated allele frequencies, a burn‐in of 100,000 iterations and subsequently 500,000 iterations to calculate the likelihood of the different models. We performed five replicate runs for each *K* ranging from *k* = 1 to *k* = 5. The number of clusters was determined according to the MedMeaK and MaxMeaK method (Puechmaille, 2016) with a threshold of 60% for the mean membership coefficient. The results were illustrated using DISTRUCT (Rosenberg, 2004). In addition to the structure analysis, clustering methods implemented in InStruct (Gao, Williamson, & Bustamante, 2007) and Flock (Duchesne & Turgeon, 2012) were investigated. The Bayesian clustering method of InStruct inferred the number of subpopulations only with admixture by comparing the log likelihoods and deviance information criterion (DIC) for the number of subpopulations ranging from *K* = 2 to *K* = 5. For that purpose, samples were taken every 100 iterations from three independent chains with 1,000,000 iterations and 500,000 iterations as burn‐in. Convergence was checked with Gelman‐Rudin statistics. In Flock, the plateau lengths were determined for two to nine reference groups using 30 iterations and 50 runs with a random choice of samples as the initial separation mode. Furthermore, identical multi‐locus genotypes were identified using GenClone2 (Arnaud‐Haond & Belkhir, 2007). Accordingly, we removed 107 individuals so that only one copy of each genotype is present per sample. The analyses of population structure were repeated with the purged samples. However, as the results were similar to the analyses including all individuals and purging might reduce precision of the fixation index (Waples & Anderson, 2017), we only show results of the analyses including all individuals.

### 2.6 Comparing genotype with cohort, sex, and environmental data

Individuals were assigned to a distinct genetic cluster defined by Structure when membership to that cluster was higher than 60%. Choosing higher membership thresholds resulted in an increase of unassigned individuals, but did not change the trends. Additionally, when possible, individuals used in this study were assigned to distinct cohorts based on their size (Thonig et al., 2016). We analyzed whether the different size cohorts are composed of individuals assigned to distinct genetic clusters. Similarly, as some individuals were identified as bearing gametes, we analyzed whether these females and males were assigned to different genetic clusters. We estimated genetic differentiation (1) between different cohorts within each site, (2) of females/males between every sampling within each site, and (3) between males, females, and all individuals within each sample, using the fixation index $G_{\mathrm{ST}}^{\prime }$ (Hedrick, 2005) as implemented in the R package diveRsity (Keenan et al., 2013). This was only applicable when more than one specimen was present per group and more than two groups were present for comparison.

We compared the observed genetic structure at four time points (March, May, August, and November) with the environmental parameters described previously (Thonig et al., 2016) using Primer‐E v.6 (Clarke & Gorley, 2006). For that purpose, genetic differentiation $G_{\mathrm{ST}}^{\prime }$ calculated with the R package diveRsity (Keenan et al., 2013) was input in Primer‐E as a dissimilarity matrix. The following environmental parameters were normalized and used to calculate a resemblance matrix based on Euclidian distance: median particle size (correlating significantly with sorting *r* = −.818, porosity *r* = .725, and water content *r* = .775), organic content, C/N, mean temperature (correlating significantly with standard deviation of temperature *r* = .905), mean salinity, and standard deviation of salinity. The Spearman rank correlation between the two matrices was calculated using RELATE, and the environmental parameters best explaining the observed genetic differentiation were determined via DistLM based on the Bayesian information criterion (BIC) using 9999 permutations.

## 3 RESULTS

### 3.1 Genetic diversity

The genetic diversity of the metapopulation is described in Table 2. Allelic richness and expected heterozygosity are similar among sites throughout the year, being highest in August and October. However, this increase in diversity is less distinct in Lynæs and Herslev than at the other sites. Depending on the location, the percentage of private alleles increases from May to November and is highest at Vellerup and lowest at Lynæs. Gene diversity not only fluctuates but also seems to peak in August and October. Accordingly, relatedness is lowest in August and October, most drastically at Lammefjord and Vellerup. Gene diversity and relatedness are otherwise similar among the sites. At Vellerup and Herslev, the observed heterozygosity fluctuates through the year more than it does at the other sites. In almost all of the samples, a deficiency of heterozygotes was observed, with significant differences from HWE in the majority of samples from Lammefjord and Vellerup.

**Table 2:** Genetic diversity for each sample

Sample	*N*	*H*_e_	*H*_o_	Gene diversity	*F*_IS_	Allelic richness (*N* = 26)	Private alleles (*N* = 26)	Mean relatedness
Lynæs								
March	35	0.313	0.281	0.222	0.099	2.54	0	0.393
May	36	0.322	0.268	0.263	0.143*	3.29	0.1	0.226
August	29	0.315	0.283	0.239	0.077	4.02	0.01	0.246
October	35	0.364	0.310	0.364	0.150*	4.16	0.11	0.199
November	38	0.337	0.320	0.289	0.051	2.86	0.1	0.311
February	29	0.339	0.345	0.338	−0.021	3.34	0.0	0.231
Lammefjord								
March	31	0.330	0.347	0.276	−0.068	2.78	0	0.287
May	44	0.319	0.320	0.264	−0.022	3.07	0	0.344
August	41	0.416	0.348	0.416	0.165*	5.71	0.21	0.131
October	30	0.414	0.346	0.351	0.142*	5.2	0.13	0.119
November	40	0.335	0.291	0.321	0.112*	4.33	0	0.184
February	40	0.371	0.301	0.366	0.183*	4.42	0.11	0.194
Vellerup								
March	32	0.355	0.372	0.300	−0.056	2.52		0.297
May	37	0.330	0.251	0.299	0.226*	3.75	0.33	0.231
August	27	0.400	0.349	0.388	0.115*	5.51	0.37	0.147
October	37	0.427	0.344	0.415	0.182*	5.65	0.58	0.140
November	33	0.333	0.262	0.276	0.201*	3.64	0	0.295
February	39	0.321	0.313	0.316	0.014	3.04	0	0.245
Herslev								
March	41	0.318	0.280	0.314	0.112*	2.85	0.04	0.287
May	37	0.369	0.330	0.192	0.085	2.5	0.07	0.320
August	34	0.429	0.421	0.358	0.003	3.41	0.03	0.216
October	30	0.400	0.341	0.393	0.141*	3.89	0.06	0.189
November	43	0.336	0.309	0.283	0.072	3.09	0.3	0.272
February	37	0.350	0.352	0.284	−0.025	3.22	0	0.202

*N*, number of individuals per sample.

Expected and observed heterozygosity (*H*_e_ and *H*_o_), gene diversity, and inbreeding coefficient (*F*_IS_) were calculated using Arlequin v.3.5.2. *F*_IS_ values with a *p*‐value smaller than .05 are indicated with *. Allelic richness and number of private alleles were determined with HP‐Rare v1.1. Relatedness was calculated using Coancestry v.1.

### 3.2 Population structure

The AMOVA results of the temporal and spatial differences between the samples are shown in Table 3. When samples are grouped according to location (across time), a similar percentage of the variation is explained by location (1.99%) and time point within location (2.05%). When samples are grouped according to time point (across locations), a greater percentage of variation is explained by location within time points (3.17%) than among time points (0.51%). These results suggest that the four locations are genetically differentiated. Moreover, the results suggest that there is no general seasonal pattern in the population structure common to all locations; instead, temporal genetic changes differ among the locations.

**Table 3:** Analysis of molecular variance (AMOVA) of temporal and spatial groups of samples performed with Arlequin v.3.5.2

Source of variation	*df*	Sum of squares	Variance components	Percentage of variation	*p*‐Value
Spatial groups (across time)					
Among locations	3	39.073	0.02349	1.99	<.0001
Among time points within locations	20	59.26	0.02425	2.05	<.0001
Among individuals within sample	831	1030.743	0.10527	8.9	<.0001
Among loci within individuals	855	880.5	1.02982	87.06	<.0001
Temporal groups (across locations)					
Among time points	5	28.308	0.006	0.51	.11517
Among locations within time points	18	70.025	0.03735	3.17	<.0001
Among individuals within sample	831	1030.743	0.10527	8.93	<.0001
Among loci within individuals	855	880.5	1.02982	87.38	<.0001

**Figure 1:**
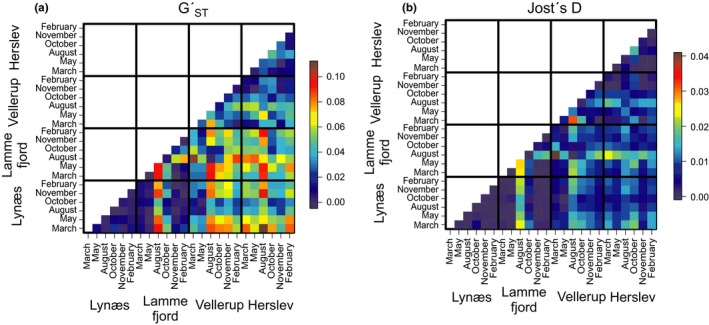
Heatmap illustrating the differentiation between the sampled populations using the fixation index $G_{\mathrm{ST}}^{\prime }$ (panel a) and Jost′s D (panel b) calculated with diveRsity, ranging from genetically similar populations in blue (0.00) to genetically differentiated populations in red (up to 0.1). The respective confidence intervals can be found in Table S1

The two summary statistics for population differentiation, $G_{\mathrm{ST}}^{\prime }$ and Jost′s D (Figure 1), show similar patterns, but Jost′s D shows less pronounced differentiation between the samples. Except in August, Herslev is more similar to Vellerup, whereas Lammefjord and Lynæs are more alike in allele frequencies, but these two groups differ from each other. There are no seasonal differences in samples from Lynæs, and only weak differences among the samples from Herslev. However, strong seasonality occurs at Lammefjord and Vellerup. Allele frequencies in August, and to some degree also in October, differ from those in the other months at these sites and also differ from allele frequencies at other locations. However, allele frequencies in August and October at Lammefjord and Vellerup are similar. The confidence intervals of $G_{\mathrm{ST}}^{\prime }$ and Jost′s D can be found in Table S1.

**Figure 2:**
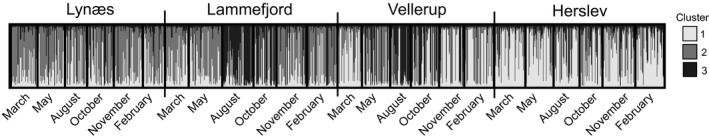
Assignment of sampled individuals to different genetic clusters as determined with the program Structure for *K* = 3 clusters. Each line represents one individual sampled from four locations at six different months in chronological order from left to right. The color of the line describes the membership of that individual to the three respective clusters. Cluster 1 (light gray) was composed of samples from Herslev in March, May, *August*,* October*, November, and *February*; and from Vellerup in March, November, and *February*. Cluster 2 (gray) was composed of samples from Lynæs in March, *May*,* August*,* October*, November, and *February*; from Lammefjord in *March*, May, *November*, and February; and from Vellerup in *May*. Cluster 3 (dark gray) was composed of samples from Lammefjord in August and *October*; and from Vellerup in August and *October*. If the average membership in one cluster for a sample was less than 60%, the sample is listed in italic

The cluster analysis in Structure revealed three genetic clusters when analyzing the whole metapopulation (Figure 2). The first cluster (light gray) was composed of all samples from Herslev as well as samples from March, November, and February from Vellerup. The second cluster (gray) included all samples from Lynæs, all samples from Lammefjord except for August and October, and the sample from May from Vellerup. The third cluster (dark gray) contained the samples from August and October from both Lammefjord and Vellerup. For *k* = 3, the allele frequency divergence among clusters computed by Structure using point estimates is lower between the first and second cluster (0.0339) compared to the divergence of the third cluster from the other two (first to third 0.0509, second to third 0.0749). Analyzing every location separately resulted in a single cluster for both Herslev and Lynæs, whereas two clusters were the best solution for the samples at Lammefjord and Vellerup (graphs not shown). In both cases, the first cluster included the samples from March, May, November, and February, while the samples from August and October belonged to the second cluster.

Genetic clusters were also estimated with the program InStruct, which accounts for inbreeding and might be more suitable for *P. elegans*, because we observed high and significant *F*_IS_ values and *P. elegans* also is able to reproduce asexually. This program recommended two clusters as a best explanation for the data according to deviance information criterion (DIC). The program Flock determines genetic clusters by partitioning the sample and reallocating genotypes. Several runs starting with a different initial partitioning are performed for a different number of clusters. The number of genetic clusters *k* is reached when an identical final partitioning is obtained for more than six runs. For *k* = 2–9 cluster, no more than three identical partitions were obtained for our sample, indicating that either no population structure is present or our data do not contain enough information, that is, too few microsatellites, to infer the number of genetic clusters. Of the three methods used to estimate genetic clusters, the three‐cluster solution from Structure reflects the $G_{\mathrm{ST}}^{\prime }$ values best.

### 3.3 Comparing genotype with cohort, sex, and environmental data

**Figure 3:**
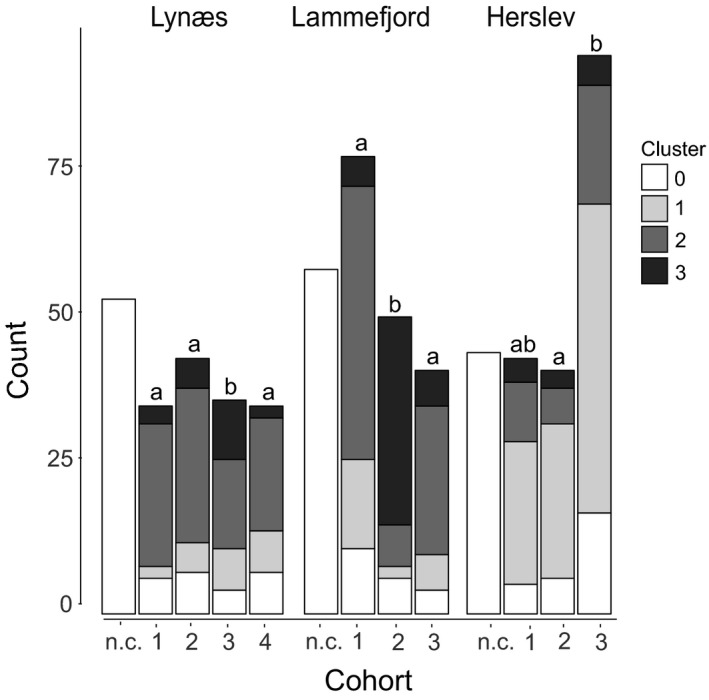
Assignment of individuals to different size cohorts (according to Thonig et al., 2016) and genetic clusters (1, 2, or 3) as determined with Structure when membership coefficient was larger than 60%. Cluster 0 indicates individuals that could not be assigned to a distinct genetic cluster (membership coefficient was less than 60%). Size cohort is listed in the *x*‐axis, n.c. groups those individuals that could not be assigned to a size cohort because of overlapping size ranges and for that reason were not assigned to a genetic cluster. No size cohorts could be distinguished at Vellerup; hence, it was excluded from this graph. Genetic differentiation between cohorts within site was tested using $G_{\mathrm{ST}}^{\prime }$ and is indicated with lowercase letters. The respective values and confidence intervals can be found in Table S2

Cohorts based on size of the worms were distinguished previously (Thonig et al., 2016), and the genetic composition of these cohorts is shown in Figure 3 and Table S2. Vellerup is not included, as we could not distinguish cohorts based on size at this location (see Thonig et al., 2016 for more details). At the other sites, about 50 individuals per site could not be assigned to a distinct size cohort due to overlapping size ranges of the cohorts (cohort not characterized—n.c.). About 25 individuals per site that could be assigned to a cohort, however, could not be assigned to a distinct genetic cluster as their membership coefficient was below 60% (cluster 0). At Herslev, the fixation index *G*’_ST_ suggests a genetic difference between cohort 2 and 3 (*G*’_ST_  = 0.0138). Most individuals in the cohorts at Herslev were assigned to genetic cluster 1 (68%), but some individuals in the size cohorts were assigned to genetic cluster 2 (23%) and genetic cluster 3 (9%). Likewise, at Lynæs, all four size cohorts are primarily composed of individuals assigned to a single genetic cluster: cluster 2 (67%). Nonetheless, individuals assigned to the other two genetic clusters also exist and vary in frequency. Note that 31% of the individuals of the third size cohort belong to genetic cluster 3, whereas individuals assigned to genetic cluster 3 make up only 7%–14% of the other size cohorts. A significant *G*’_ST_ value indicates that cohort 3 differs from all other cohorts (*G*’_ST_ ranging from 0.0124 to 0.0243). In contrast to relatively stable genetic composition of size cohorts in Herslev and Lynæs, Lammefjord shows a different pattern: the first and third size cohorts are dominated by individuals assigned to genetic cluster 2 (~70%), while the second size cohort is dominated by individuals assigned to genetic cluster 3 (80%). The genetic difference between size cohorts is evidenced by significant *G*’_ST_ values between cohorts (*G*’_ST_ ranging from 0.0694 to 0.0826). These patterns reflect the seasonal variation noted in the initial structure analysis (Figure 2). The third size cohort at Lynæs and the second size cohort at Lammefjord, which show higher frequencies of individuals assigned to genetic cluster 3, are both present from June to October/November. The second cohort at Herslev that differed slightly from the other two cohorts due to less individuals assigned to cluster 2 and more individuals assigned to cluster 1 was present during the whole study period, but dominated from June to September.

**Figure 4:**
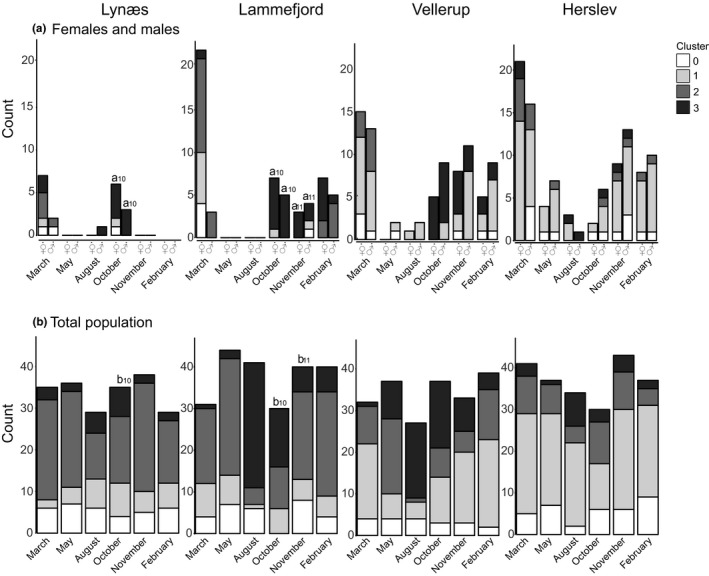
Assignment of females and males (a) as well as the total population (b) to the three genetic clusters (1, 2, or 3) determined with Structure when membership coefficient was larger than 60%, at each time point and site. Females and males were identified by the presence of eggs or sperm in their coelom, respectively. Cluster 0 indicates individuals that could not be assigned to a distinct genetic cluster (membership coefficient was less than 60%). Genetic differentiation among females, males, and the total sample was tested using $G_{\mathrm{ST}}^{\prime }$, and significant differences are indicated with lowercase letters. No statistics could be calculated when less than two individuals were in one sample or when there were less than three samples per comparison. Hence, no results are available for within sample comparisons for Lynæs except in October, for Lammefjord and Vellerup in May and August, and for Herslev in August. The respective values and confidence intervals can be found in Tables S3 and S4

In Figure 4 and Tables S3 and S4, the genetic composition of the individuals bearing gametes are shown (panel a) in comparison with the genetic composition of the whole sample (panel b). Individuals with membership coefficients below 60% are excluded (cluster 0). At Herslev, we did not observe a significant genetic change among individuals with eggs or sperm between samplings: These individuals mostly belong to genetic cluster 1 throughout the study period. At the other sites, individuals reproducing in winter/spring are primarily assigned to genetic clusters 1 and 2, whereas individuals reproducing in fall/winter are primarily assigned to genetic cluster 3. However, at Vellerup, individuals assigned to genetic cluster 1 are also reproductive in fall. While too few sexually mature individuals were captured at Lynæs to test for genetic differences, at Lammefjord and Vellerup, gravid females and ripe males sampled in March showed genetic differences from those sampled in October and November, and at Lammefjord, gravid females and ripe males sampled in October also differed genetically from those sampled in February.

Comparing the genetic composition of reproductive individuals and that of all individuals in each sample, we can see whether individuals contributing to the next generation represent a subsample of the available genetic variation (see Figure 4 and Table S4). At Herslev, the percentages of individuals in the different genetic clusters among reproductive females and males are similar to those of the whole population, with genetic cluster 1 dominating both. This is further supported by the fact that we observed no significant $G_{\mathrm{ST}}^{\prime }$ value between reproductive individuals and the whole sample. At the other sites, discrepancies are seen from October to February, when genetic cluster 3 is more common among reproductive females and males even though in the total population a different genetic cluster is more prevalent: cluster 2 at Lynæs in October and at Lammefjord in February, or cluster 1 at Vellerup in November. At Vellerup, no individuals with gametes belong to cluster 2 in fall even though individuals in cluster 2 are relatively common in the population. Likewise, significant genetic differentiation was observed between females/males and the whole sample at Lynæs in October and at Lammefjord in October and November. No differentiation was observed at Vellerup, however, probably because of the genetic similarity of clusters 1 and 2.

We observed a significant moderate correlation (ρ = 0.4, *p* = .001) between genetic and environmental differences between samples. The environmental parameters best correlating with the genetic differentiation and explaining 74.02% of the genetic variation are median grain size, mean temperature, and mean salinity. These results are displayed in a distance‐based redundancy analysis (dbRDA) in Figure S1.

## 4 DISCUSSION

We investigated the genetic structure of the poecilogonous polychaete *P. elegans* from four sites in the Isefjord‐Roskilde‐Fjord estuary complex using six temporal samples collected over 1 year. We aimed to evaluate the relationship between the genotype of sampled individuals and previously described differences in population dynamics in these populations (see Thonig et al., 2016). We observed genetic differences between the sites as well as changes during the year at two of the sites. Similar population genetic structure was evident from summary statistics and fixation indices, cluster analysis, and AMOVA. Overall, differentiation is low, which we expected given that the sites are geographically close and that the time between sampling is short. Nevertheless, significant genetic differentiation was found between cohorts as well as between reproductive individuals and the total population. Previously, Kesäniemi, Hansen, Banta, and Knott (2014) also detected three genetically different clusters among 16 sampling sites within the Isefjord‐Roskilde‐Fjord estuary complex at a single time point in 2010. Furthermore, Kesäniemi, Mustonen, Boström, Hansen, and Knott (2014) found either temporal stability or differences in allele frequencies, depending on the population, when sampling different populations in Baltic Sea to North Sea over 1–2 years. However, neither of these previous studies included sufficient sampling and phenotypic data to allow the assessment of genetic composition of size cohorts or reproducing individuals.

### 4.1 Seasonal dynamics

The genetic data collected in this study suggest the arrival of genetically distinct recruits of *P. elegans* after May and after October at Lammefjord and Vellerup, indicated by a temporal change of the predominant genetic cluster. The timing of the genetic shift at these sites correlates with the appearance of new cohorts defined by size (Thonig et al., 2016). Accordingly, small individuals (<30 setigers) appeared at the four study sites in spring (April to June) and in autumn (September to November), and individuals died in summer (July) and winter (January) (Thonig et al., 2016). Similar recruitment times in spring and fall have been reported for other populations of *P. elegans* (Bolam, 2004; Gudmundsson, 1985; Morgan, 1997). Moreover, the second size cohort at Lammefjord and Herslev as well as the third size cohort at Lynæs was composed of genetically different individuals compared to the other cohorts at this site (Figure 3). As we were unable to distinguish size cohorts at Vellerup, we cannot say whether the observed seasonal genetic switch represents different size cohorts, but we assume this to be the case.

The occurrence of genetically distinct clusters in Lammefjord and Vellerup, correlated with arrival of new recruits and expected seasonal reproductive periods, suggests that these individuals immigrated from a genetically differentiated, but unknown, source population. Considering the structure of the estuary, the source of the recruits might be located within Isefjord, in close proximity to Lammefjord and Vellerup. It is also possible that larvae immigrated from the Kattegat, outside the estuary. Although the recruiting individuals were assigned to the same genetic cluster, it is important to keep in mind that they might not have originated from the same source population, as population structure in this estuarine system is known to be patchy (Kesäniemi, Hansen et al., 2014). Kesäniemi, Hansen et al. (2014) also included samples from Lammefjord and Vellerup in their broader spatial study of samples collected at a single time point (April 2010), but in that study, the two populations were assigned to different genetic clusters. Our analysis indicates some differentiation between the populations in spring as well, despite their genetic similarities in the fall. At least in March, the sample from Vellerup was assigned to cluster 1 while the sample from Lammefjord was assigned to cluster 2, but in May, individuals from both populations were primarily grouped in cluster 2. In contrast to what was observed at Lammefjord and Vellerup, we could not detect any seasonal genetic change at Lynæs and Herslev in the structure analysis, indicating that the majority of new recruits at these locations did not originate from differentiated populations or are the result of self‐recruitment. However, the presence of some immigrants belonging to the third genetic cluster at these two sites might also reflect presettlement selection due to dispersal limitation or missing habitat cues.

Along with the seasonal genetic change noted for populations at Lammefjord and Vellerup, we observed that the reproductive individuals also show a genetic change at these sites, and surprisingly, also at Lynæs, where population‐level seasonal variation was not detected. Gamete‐bearing individuals were assigned primarily to genetic clusters 1 and 2 in winter to spring, but assigned primarily to cluster 3 in fall, and persisting partly in winter. This pattern correlates with the two peaks of gravid females and ripe males, in September/October and in January/February that we observed at these sites (Thonig et al., 2016). In contrast, only a single peak of individuals with gametes was noted at Herslev, and here, individuals were primarily assigned to cluster 1 during the whole period. Hereby, the genetic change in females is particularly of interest as they can store sperm in *receptacula seminis* and so the contribution of ripe males to the next generation is not clear.

In our previous study (Thonig et al., 2016), we examined egg strings produced in these populations in order to determine larval developmental mode and found that at Lammefjord, Lynæs, and Vellerup, planktonic larvae were produced primarily from November to February, but benthic and intermediate larvae were produced primarily from February to June. At Herslev, only benthic and intermediate larvae were predominant throughout the reproductive period (November to May). Seasonal switches in developmental mode that we observed (Thonig et al., 2016) have also been noted by others (Gudmundsson, 1985 and Rasmussen, 1973) and might indicate asynchronous local population dynamics (isolation by time) where gene flow is restricted due to reproductive season (Eldon, Riquet, Yearsley, Jollivet, & Broquet, 2016; Hendry & Day, 2005). Such dynamics were noted for the polychaete *Pectinaria koreni* in Baie de Seine (Jolly, Thiébaut, Guyard, Gentil, & Jollivet, 2014). With asynchronous population dynamics, we would expect to observe genetic changes between seasons, but not years (Hendry & Day, 2005). The results of this study show seasonal genetic change at some sites, but not others. Although our sampling did not cover multiple years, Kesäniemi, Mustonen et al. (2014) observed temporal genetic change at Vellerup between spring 2009 and spring 2010, but no genetic change between fall 2008 and spring 2009/2010, in other words, annual but not seasonal genetic change. Together, these results highlight temporal variation with no clear pattern that even can differ among geographically close populations.

### 4.2 Chaotic genetic patchiness

Although we analyzed populations at only four sites located in close proximity in the same estuary, we observed different genetic clusters and different seasonal dynamics among them, confirming chaotic genetic patchiness (CGP) among *P. elegans* in the Isefjord‐Roskilde‐Fjord estuary complex as reported by Kesäniemi, Hansen et al. (2014). Chaotic genetic patchiness describes spatial genetic structure with high temporal turnover at a scale where dispersal should be able to efficiently homogenize genetic variation (Eldon et al., 2016). One likely mechanism of CGP is sweepstakes reproductive success (SRS), the variance in reproductive success of highly fecund marine organisms and unequal contributions to the future reproductive population due to the high degree of stochasticity of oceanographic processes, spawning success, and fates of planktonic larvae (Broquet, Viard, & Yearsley, 2013; Cornwell, Fisher, Morgan, & Neigel, 2016; Hedgecock, 1994; Hedgecock & Pudovkin, 2011). Although the reproductive biology of *P. elegans* does not match that of species for which SRS is described originally, our analysis indicates that reproductive individuals are not necessarily a random subset of the population: Genetic cluster 2 is present at all sites, but these individuals do not contribute to reproduction in respective proportions. Moreover, at Vellerup and Herslev, maximum 40%–60% of the individuals carry gametes while only 20%–40% of the population are reproductive at Lammefjord and Lynæs (Thonig et al., 2016). Hence, SRS might be more likely at the latter sites, in particular at Lynæs, where reproducing individuals do not belong to the dominant genetic cluster. The effect of SRS can be counterbalanced via larval dispersal that redistributes genetic variation between locations (Eldon et al., 2016). However, if larvae from different populations are not well‐mixed, but instead disperse together with others from the same cohort, termed collective dispersal, the effect of genetic drift due to small effective population size will be maintained (Broquet et al., 2013; Eldon et al., 2016). Our observations of a change in the predominate genetic cluster with the appearance of a new size cohort suggest that collective dispersal could occur, but additional study of larval cohorts and their genetics is needed to support this hypothesis. The short life span of *P. elegans* and its seasonal reproduction also likely enhance the consequences of SRS.

Diversifying selection is another mechanism that can cause CGP in unstable and patchy environments (Eldon et al., 2016). Therefore, poecilogony of *P. elegans* alone (without SRS or collective dispersal) might explain the patterns of chaotic genetic patchiness we observed in the Isefjord‐Roskilde‐Fjord estuary complex. Heterogeneous environments can promote the evolution of phenotypic polymorphisms, depending on the accuracy of both genetic and environmental cues that influence development of the phenotype (Leimar, 2009) and are expected to favor the evolution of poecilogony (Chia, Gibson, & Qian, 1996). For example, when environmental heterogeneity is unpredictable, diversifying bet‐hedging within cohorts might explain observed variation in developmental mode (Krug, 2009). In this and our previous study, the environmental parameters explaining best the population genetic structure and population dynamics, respectively, were temperature, sediment grain size correlating with sorting, and mean salinity (Thonig et al., 2016), and these variables likely describe different aspects of the environmental heterogeneity. Temperature reflects the seasonal changes in reproductive activity and population genetic structure observed in Lammefjord and Vellerup. However, the populations that did not show seasonal genetic changes also experienced seasonal fluctuations in temperature. The coarse and poorly sorted sediment at Vellerup and Herslev was inhabited by large specimens of *P. elegans*, and populations showed high densities as well as a high percentage of gamete‐bearing individuals. These populations were additionally the ones where most individuals were assigned to the first genetic cluster (38% at Vellerup and 55% at Herslev). In contrast, at Lynæs and Lammefjord, the sediment was fine and well‐sorted, and specimens were smaller, occurred in lower densities, and had higher percentage of asexual reproduction. These populations were dominated by the second genetic cluster (57% at Lynæs and 47% at Lammefjord). Salinity was only lower at Herslev (~15 PSU) compared to the other sites (~20 PSU). Herslev was also the only site where no switch in developmental mode and genetic composition of reproductive individuals was observed. Other environmental variables not measured here might be more effective in explaining the patterns of genetic differentiation we observed here.

Zakas and Hall (2012) proposed dispersal polymorphism in *Streblospio benedicti* between similar sized patches is maintained due to asymmetric dispersal, as in typical source‐sink metapopulations. Source‐sink metapopulations are composed of several subpopulations with heterogeneous habitat quality. Demographic excess in high‐quality habitats (sources) can lead to emigration, while low‐quality habitats (sinks) with a demographic deficit might not persist without immigration, can go extinct, and be recolonized (Dias, 1996). If such is the case also for *P. elegans*, the different population dynamics we observed suggest that Herslev and Vellerup would be characterized as source subpopulations, while Lynæs and Lammefjord would be sinks, according to criteria described by Jolly et al. (2014). However, De Meester, Gómez, Okamura, and Schwenk (2002) and Jolly et al. (2014) proposed that genetic heterogeneity and temporal change are high and allelic richness low in sink populations and vice versa in sources. This is only partly the case for populations in our study. Hence, the heterogeneous, unpredictable character of the estuary and metapopulation dynamics might maintain poecilogony in *P. elegans* as a bet‐hedging strategy in the Isefjord‐Roskilde‐Fjord estuary complex in comparison with other sites where *P. elegans* are expected to be fixed to a certain mode of development (Bolam, 2004; Morgan et al., 1999).

## 5 CONCLUSION

We found spatial and seasonal population genetic structure of *P. elegans* in the Danish Isefjord‐Roskilde‐Fjord estuary complex, but seasonal genetic structure varied among the four study sites. When present, the seasonal genetic switch correlated with the arrival of new size cohorts. Phenotypic variation in larval developmental mode of *P. elegans* contributes to patterns of chaotic genetic patchiness observed in the estuary metapopulation. We found that the genotypes of individuals bearing gametes did not resemble the genotypes of the whole sample, indicating a possibility for variance in reproductive success. However, the genetics of larval cohorts and the effects of pre‐ and postlarval settlement on the population genetics are yet to be determined. Diversifying selection could lead to poecilogony in *P. elegans* as a bet‐hedging strategy to allow persistence in the unpredictable estuarine environment resulting in chaotic genetic patchiness among populations.

## DATA ACCESSIBILITY

Microsatellite loci described in this study are available in GenBank under accession numbers MG021816 ‐ MG021822; GU321899, GU321900, and GU321906. Genotype and phenotype data for *Pygospio elegans* analyzed in this study are available from the Dryad Digital Repository: http://dx.doi.org/10.5061/dryad.9c5s0 .

## CONFLICT OF INTEREST

None declared.

## AUTHOR CONTRIBUTIONS

AT carried out the field work, phenotyping, genotyping, and data analysis and drafted the manuscript. KEK optimized the molecular markers and participated in data analysis and preparation of the manuscript. KEK, GTB, and BWH designed and coordinated the study. All authors made contributions to the final manuscript and approval for publication.
