# Supplementary material for: Seasonal genetic variation associated with population dynamics of a poecilogonous polychaete worm
Source: Ecol Evol. 2017 Oct 22;7(23):10005–17. doi: 10.1002/ece3.3518 (PMC5723597; doi:10.1002/ece3.3518)
Supplement: Supplementary file 1 [file ECE3-7-10005-s001.docx]

Supplementary Table S1: Population differentiation estimated with G’_ST_ and Jost´s D and their respective confidence intervals (CI) calculated with the R package diveRsity. Values are significant when 0 is not included in the CI and are displayed in bold.

|  | G’_ST_ | | | Jost´s D | | |
| --- | --- | --- | --- | --- | --- | --- |
| populations | value | lower CI | upper CI | value | lower CI | upper CI |
| H03 vs H05 | 0.0026 | -0.0175 | 0.0297 | 0 | -0.0019 | 0.0035 |
| H03 vs H08 | **0.0262** | 0.0081 | 0.0489 | 0.0037 | -0.0035 | 0.0104 |
| H03 vs H10 | 0.0076 | -0.0047 | 0.0218 | 0.0007 | -0.0035 | 0.0064 |
| H03 vs H11 | 0.0108 | -0.001 | 0.0286 | 0.0001 | -0.0025 | 0.0027 |
| H03 vs H02 | 0.0102 | -0.0035 | 0.0386 | 0 | -0.0033 | 0.0046 |
| H03 vs L03 | **0.0437** | 0.0189 | 0.0719 | **0.0062** | 0.0026 | 0.0113 |
| H03 vs L05 | **0.0663** | 0.0418 | 0.0944 | **0.0107** | 0.0068 | 0.0162 |
| H03 vs L08 | **0.0802** | 0.0496 | 0.0979 | **0.0242** | 0.014 | 0.0337 |
| H03 vs L10 | **0.0529** | 0.0258 | 0.0727 | **0.011** | 0.0041 | 0.0151 |
| H03 vs L11 | **0.0474** | 0.024 | 0.0663 | **0.0074** | 0.0034 | 0.0131 |
| H03 vs L02 | **0.0522** | 0.0316 | 0.0719 | **0.0076** | 0.0041 | 0.0104 |
| H03 vs U03 | **0.0832** | 0.0558 | 0.1134 | **0.0141** | 0.0093 | 0.0217 |
| H03 vs U05 | **0.067** | 0.0477 | 0.0882 | **0.0116** | 0.0067 | 0.018 |
| H03 vs U08 | **0.0425** | 0.017 | 0.0904 | **0.0081** | 0.0033 | 0.0133 |
| H03 vs U10 | **0.0283** | 0.0118 | 0.0553 | **0.0054** | 0.0009 | 0.0105 |
| H03 vs U11 | **0.0573** | 0.0385 | 0.0959 | **0.0088** | 0.0058 | 0.0116 |
| H03 vs U02 | **0.0571** | 0.0255 | 0.0745 | **0.0105** | 0.0037 | 0.0173 |
| H03 vs U02 | 0.0134 | -0.001 | 0.0346 | 0.0026 | -0.0021 | 0.0093 |
| H03 vs V05 | **0.0295** | 0.0052 | 0.0717 | 0.0049 | 0 | 0.0155 |
| H03 vs V08 | **0.059** | 0.0263 | 0.0822 | **0.0185** | 0.0011 | 0.0335 |
| H03 vs V10 | 0.0275 | -0.0029 | 0.044 | 0.0088 | -0.0034 | 0.0175 |
| H03 vs V11 | 0.0068 | -0.0042 | 0.0215 | 0.0001 | -0.0026 | 0.0039 |
| H03 vs V02 | 0.0128 | -0.0061 | 0.0357 | 0.0024 | -0.0012 | 0.0067 |
| H05 vs H08 | **0.0264** | 0.0134 | 0.0494 | 0.0016 | -0.0036 | 0.0083 |
| H05 vs H10 | 0.0297 | -0.0005 | 0.0824 | **0.0089** | 0.0004 | 0.0205 |
| H05 vs H11 | -0.0012 | -0.0155 | 0.0062 | 0 | -0.0024 | 0.0017 |
| H05 vs H02 | 0.0124 | -0.0002 | 0.0367 | 0.0002 | -0.0035 | 0.0081 |
| H05 vs L03 | **0.0382** | 0.0149 | 0.0724 | **0.0046** | 0.0008 | 0.0098 |
| H05 vs L05 | **0.0527** | 0.0363 | 0.0761 | **0.0064** | 0.0037 | 0.0114 |
| H05 vs L08 | **0.0844** | 0.0569 | 0.1232 | **0.0206** | 0.0115 | 0.0383 |
| H05 vs L10 | **0.0551** | 0.0238 | 0.0852 | **0.0091** | 0.0031 | 0.0167 |
| H05 vs L11 | **0.0416** | 0.0278 | 0.0679 | **0.0052** | 0.0022 | 0.0121 |
| H05 vs L02 | **0.0617** | 0.0425 | 0.0777 | **0.0084** | 0.0052 | 0.0108 |
| H05 vs U03 | **0.064** | 0.047 | 0.0969 | **0.0077** | 0.0054 | 0.0136 |
| H05 vs U05 | **0.058** | 0.0223 | 0.0903 | **0.0077** | 0.0037 | 0.0112 |
| H05 vs U08 | **0.0364** | 0.0043 | 0.0738 | **0.0051** | 0.0006 | 0.0111 |
| H05 vs U10 | **0.0326** | 0.0103 | 0.0473 | **0.0051** | 0.001 | 0.0101 |
| H05 vs U11 | **0.0545** | 0.0394 | 0.0698 | **0.0065** | 0.0049 | 0.0079 |
| H05 vs U02 | **0.0478** | 0.0285 | 0.0771 | **0.0066** | 0.0015 | 0.0126 |
| H05 vs U02 | 0.0149 | -0.0006 | 0.0357 | 0.003 | -0.0011 | 0.0106 |
| H05 vs V05 | **0.0451** | 0.0219 | 0.0747 | **0.0068** | 0.0033 | 0.0116 |
| H05 vs V08 | **0.0608** | 0.02 | 0.1064 | **0.0153** | 0.0012 | 0.0357 |
| H05 vs V10 | **0.0371** | 0.0169 | 0.053 | **0.0089** | 0.0029 | 0.0154 |
| H05 vs V11 | 0.0002 | -0.0142 | 0.0174 | 0 | -0.0018 | 0.0032 |
| H05 vs V02 | **0.0113** | 0.0014 | 0.0356 | 0.0013 | -0.0008 | 0.0045 |
| H08 vs H10 | **0.0467** | 0.0243 | 0.0808 | **0.0168** | 0.0062 | 0.0331 |
| H08 vs H11 | **0.0308** | 0.0107 | 0.0478 | 0.0031 | -0.0037 | 0.0116 |
| H08 vs H02 | **0.0367** | 0.011 | 0.0539 | 0.0043 | -0.0086 | 0.0141 |
| H08 vs L03 | **0.0888** | 0.0522 | 0.1199 | **0.0132** | 0.0062 | 0.02 |
| H08 vs L05 | **0.0921** | 0.0712 | 0.1184 | **0.0132** | 0.0086 | 0.0225 |
| H08 vs L08 | **0.0687** | 0.0382 | 0.1066 | **0.0172** | 0.0102 | 0.0247 |
| H08 vs L10 | **0.0502** | 0.0208 | 0.0837 | **0.0084** | 0.0037 | 0.0124 |
| H08 vs L11 | **0.0761** | 0.0432 | 0.1075 | **0.0112** | 0.0062 | 0.0168 |
| H08 vs L02 | **0.094** | 0.0597 | 0.1268 | **0.0139** | 0.009 | 0.0194 |
| H08 vs U03 | **0.1116** | 0.0897 | 0.1386 | **0.0165** | 0.0125 | 0.0244 |
| H08 vs U05 | **0.0701** | 0.0366 | 0.1039 | **0.009** | 0.0047 | 0.0129 |
| H08 vs U08 | **0.059** | 0.0252 | 0.0896 | **0.0093** | 0.0038 | 0.0161 |
| H08 vs U10 | **0.0519** | 0.0284 | 0.0728 | **0.0087** | 0.0041 | 0.0151 |
| H08 vs U11 | **0.0985** | 0.0629 | 0.1334 | **0.0141** | 0.0086 | 0.0202 |
| H08 vs U02 | **0.0771** | 0.05 | 0.1053 | **0.0119** | 0.0059 | 0.0189 |
| H08 vs U02 | **0.0373** | 0.0114 | 0.067 | 0.0077 | -0.0011 | 0.0208 |
| H08 vs V05 | **0.0685** | 0.0488 | 0.0861 | **0.0116** | 0.0092 | 0.0141 |
| H08 vs V08 | **0.0331** | 0.0056 | 0.0568 | **0.008** | 0.0002 | 0.0162 |
| H08 vs V10 | **0.031** | 0.0114 | 0.0505 | **0.0089** | 0.0023 | 0.0145 |
| H08 vs V11 | **0.0261** | 0.0094 | 0.0465 | 0.0039 | -0.0034 | 0.0119 |
| H08 vs V02 | **0.0329** | 0.0058 | 0.0638 | 0.0052 | -0.0003 | 0.0128 |
| H10 vs H11 | **0.0292** | 0.0013 | 0.0632 | 0.0027 | -0.0008 | 0.0089 |
| H10 vs H02 | 0.0077 | -0.0128 | 0.0267 | 0.0011 | -0.0031 | 0.0072 |
| H10 vs L03 | 0.0395 | -0.003 | 0.0805 | 0.0104 | -0.0013 | 0.0191 |
| H10 vs L05 | **0.0637** | 0.0098 | 0.0966 | **0.0191** | 0.003 | 0.0284 |
| H10 vs L08 | **0.0479** | 0.0104 | 0.116 | **0.0151** | 0.0035 | 0.0418 |
| H10 vs L10 | 0.0182 | -0.0302 | 0.0663 | 0.0046 | -0.0086 | 0.0191 |
| H10 vs L11 | 0.026 | -0.008 | 0.0556 | 0.0063 | -0.0029 | 0.0189 |
| H10 vs L02 | 0.027 | -0.0077 | 0.0583 | 0.0047 | -0.0047 | 0.0127 |
| H10 vs U03 | **0.0769** | 0.0224 | 0.112 | **0.02** | 0.0057 | 0.0306 |
| H10 vs U05 | **0.0567** | 0.0033 | 0.1016 | **0.0165** | 0.0001 | 0.0332 |
| H10 vs U08 | **0.0358** | 0.0025 | 0.0855 | **0.0107** | 0.0016 | 0.0234 |
| H10 vs U10 | 0.0174 | -0.0119 | 0.0519 | 0.006 | -0.0021 | 0.0147 |
| H10 vs U11 | **0.0451** | 0.0098 | 0.0843 | **0.0106** | 0.0014 | 0.0197 |
| H10 vs U02 | 0.037 | -0.0104 | 0.0759 | 0.0111 | -0.0008 | 0.022 |
| H10 vs U02 | **0.0416** | 0.0172 | 0.0663 | **0.0129** | 0.0024 | 0.0241 |
| H10 vs V05 | 0.0222 | -0.0166 | 0.063 | 0.0043 | -0.0053 | 0.0164 |
| H10 vs V08 | **0.0507** | 0.0118 | 0.0917 | 0.0117 | 0 | 0.0204 |
| H10 vs V10 | 0.0143 | -0.0123 | 0.0406 | 0.0048 | -0.0054 | 0.0118 |
| H10 vs V11 | **0.0323** | 0.0018 | 0.0683 | **0.0089** | 0.0006 | 0.0187 |
| H10 vs V02 | **0.0353** | 0.0069 | 0.0704 | **0.0095** | 0.0037 | 0.0174 |
| H11 vs H02 | 0.0069 | -0.0107 | 0.0186 | 0 | -0.0032 | 0.0046 |
| H11 vs L03 | **0.0378** | 0.0102 | 0.0715 | **0.0055** | 0.001 | 0.0124 |
| H11 vs L05 | **0.0453** | 0.021 | 0.0879 | **0.0063** | 0.0026 | 0.0139 |
| H11 vs L08 | **0.0668** | 0.0542 | 0.0812 | **0.0188** | 0.013 | 0.0235 |
| H11 vs L10 | **0.0384** | 0.012 | 0.054 | **0.007** | 0.0011 | 0.0115 |
| H11 vs L11 | **0.0378** | 0.0191 | 0.0607 | **0.0056** | 0.0027 | 0.0093 |
| H11 vs L02 | **0.0505** | 0.0268 | 0.0709 | **0.0081** | 0.0041 | 0.0139 |
| H11 vs U03 | **0.0448** | 0.0211 | 0.0998 | **0.0059** | 0.0019 | 0.0181 |
| H11 vs U05 | **0.0522** | 0.0177 | 0.0867 | **0.0077** | 0.0019 | 0.016 |
| H11 vs U08 | **0.0216** | 0.0034 | 0.0383 | **0.0035** | 0.0005 | 0.0063 |
| H11 vs U10 | **0.0213** | 0.0044 | 0.0328 | 0.0037 | 0 | 0.0072 |
| H11 vs U11 | **0.0387** | 0.0125 | 0.0638 | **0.0053** | 0.0022 | 0.0098 |
| H11 vs U02 | **0.0358** | 0.0137 | 0.0736 | **0.0055** | 0.0012 | 0.0133 |
| H11 vs U02 | 0.0108 | -0.0088 | 0.0301 | 0.001 | -0.0046 | 0.0079 |
| H11 vs V05 | **0.0327** | 0.0172 | 0.0564 | **0.0059** | 0.0029 | 0.0099 |
| H11 vs V08 | **0.0425** | 0.0033 | 0.0701 | 0.0135 | -0.0005 | 0.0235 |
| H11 vs V10 | **0.0301** | 0.0177 | 0.0442 | 0.0051 | -0.0022 | 0.0101 |
| H11 vs V11 | 0.0058 | -0.0008 | 0.0131 | 0.0008 | -0.001 | 0.0027 |
| H11 vs V02 | 0.0055 | -0.0087 | 0.0217 | 0.0008 | -0.0017 | 0.0032 |
| H02 vs L03 | **0.0575** | 0.0158 | 0.093 | **0.0091** | 0.002 | 0.0201 |
| H02 vs L05 | **0.0784** | 0.0524 | 0.101 | **0.0131** | 0.0092 | 0.0169 |
| H02 vs L08 | **0.0574** | 0.0297 | 0.0873 | **0.0138** | 0.0055 | 0.0211 |
| H02 vs L10 | **0.0362** | 0.0195 | 0.0666 | **0.0062** | 0.0025 | 0.0122 |
| H02 vs L11 | **0.0501** | 0.0313 | 0.0683 | **0.0078** | 0.004 | 0.0128 |
| H02 vs L02 | **0.0584** | 0.0322 | 0.0794 | **0.0091** | 0.0037 | 0.0135 |
| H02 vs U03 | **0.0832** | 0.063 | 0.1038 | **0.0136** | 0.0104 | 0.0206 |
| H02 vs U05 | **0.0784** | 0.0547 | 0.1001 | **0.0138** | 0.0081 | 0.0184 |
| H02 vs U08 | **0.0485** | 0.0111 | 0.0967 | **0.0087** | 0.0016 | 0.0151 |
| H02 vs U10 | **0.0319** | 0.0142 | 0.0562 | **0.0061** | 0.0029 | 0.0103 |
| H02 vs U11 | **0.0617** | 0.0472 | 0.0852 | **0.0093** | 0.0066 | 0.0138 |
| H02 vs U02 | **0.0482** | 0.0298 | 0.0689 | **0.0082** | 0.0047 | 0.0149 |
| H02 vs U02 | **0.0376** | 0.0216 | 0.0612 | **0.01** | 0.0028 | 0.0178 |
| H02 vs V05 | **0.0466** | 0.0247 | 0.0696 | **0.0083** | 0.0039 | 0.0164 |
| H02 vs V08 | **0.0464** | 0.0248 | 0.0747 | **0.0138** | 0.0071 | 0.0228 |
| H02 vs V10 | **0.0245** | 0.0132 | 0.039 | **0.0071** | 0.0015 | 0.0158 |
| H02 vs V11 | **0.016** | 0.0075 | 0.0342 | **0.0014** | -0.0022 | 0.0065 |
| H02 vs V02 | **0.0164** | 0.0005 | 0.0433 | **0.0029** | -0.0008 | 0.0075 |
| L03 vs L05 | -0.004 | -0.0121 | 0.0107 | -0.0006 | -0.0013 | 0.0014 |
| L03 vs L08 | **0.0924** | 0.0539 | 0.1206 | **0.0258** | 0.0142 | 0.0392 |
| L03 vs L10 | **0.0459** | 0.0114 | 0.0742 | **0.0092** | 0.0022 | 0.023 |
| L03 vs L11 | -0.0036 | -0.015 | 0.0125 | -0.0006 | -0.0022 | 0.0029 |
| L03 vs L02 | 0.005 | -0.0134 | 0.0194 | **0.0003** | -0.0038 | 0.0044 |
| L03 vs U03 | 0.0119 | -0.0103 | 0.037 | **0.0013** | -0.0022 | 0.0068 |
| L03 vs U05 | 0.0063 | -0.0038 | 0.0185 | 0 | -0.0022 | 0.0029 |
| L03 vs U08 | 0.006 | -0.016 | 0.0502 | **0.0007** | -0.003 | 0.0074 |
| L03 vs U10 | 0.0102 | -0.0066 | 0.0373 | **0.0013** | -0.0023 | 0.0071 |
| L03 vs U11 | 0.0072 | -0.0116 | 0.0437 | **0.0008** | -0.0025 | 0.0077 |
| L03 vs U02 | 0.0065 | -0.0062 | 0.0283 | 0 | -0.0025 | 0.0054 |
| L03 vs U02 | **0.0294** | 0.0132 | 0.046 | **0.0043** | 0.0008 | 0.0086 |
| L03 vs V05 | **0.0294** | 0.0157 | 0.051 | **0.0043** | 0.0013 | 0.0085 |
| L03 vs V08 | **0.094** | 0.0346 | 0.1324 | **0.0216** | 0.0027 | 0.0395 |
| L03 vs V10 | **0.0709** | 0.0412 | 0.0919 | **0.0136** | 0.008 | 0.0169 |
| L03 vs V11 | **0.0625** | 0.0255 | 0.1094 | **0.0089** | 0.0043 | 0.018 |
| L03 vs V02 | **0.0459** | 0.0217 | 0.0725 | **0.0063** | 0.0033 | 0.0103 |
| L05 vs L08 | **0.0978** | 0.0608 | 0.1299 | **0.0261** | 0.0133 | 0.0395 |
| L05 vs L10 | **0.0491** | 0.0244 | 0.0784 | **0.0101** | 0.0038 | 0.0162 |
| L05 vs L11 | 0.0018 | -0.0052 | 0.0091 | 0 | -0.0018 | 0.0015 |
| L05 vs L02 | 0.0098 | -0.0106 | 0.0262 | 0.0006 | -0.0039 | 0.0039 |
| L05 vs U03 | 0.0007 | -0.0111 | 0.0152 | 0 | -0.0016 | 0.0046 |
| L05 vs U05 | -0.0013 | -0.0111 | 0.0198 | -0.0001 | -0.0018 | 0.0038 |
| L05 vs U08 | -0.0009 | -0.017 | 0.0255 | 0 | -0.0022 | 0.0034 |
| L05 vs U10 | 0.0133 | -0.0016 | 0.0332 | 0.0021 | -0.0009 | 0.0072 |
| L05 vs U11 | 0.0037 | -0.013 | 0.028 | 0.0003 | -0.0021 | 0.0048 |
| L05 vs U02 | 0.0055 | -0.0076 | 0.0172 | 0 | -0.0021 | 0.0032 |
| L05 vs U02 | **0.0319** | 0.0175 | 0.0588 | **0.0047** | 0.002 | 0.0087 |
| L05 vs V05 | **0.0329** | 0.0082 | 0.0543 | **0.0057** | 0.0021 | 0.0096 |
| L05 vs V08 | **0.0921** | 0.0524 | 0.1244 | **0.02** | 0.0062 | 0.0351 |
| L05 vs V10 | **0.0765** | 0.0465 | 0.1052 | **0.0151** | 0.0087 | 0.0216 |
| L05 vs V11 | **0.0719** | 0.0304 | 0.1054 | **0.0101** | 0.005 | 0.0139 |
| L05 vs V02 | **0.0493** | 0.0246 | 0.0673 | **0.0067** | 0.0043 | 0.0086 |
| L08 vs L10 | 0.0043 | -0.0182 | 0.0294 | 0 | -0.0048 | 0.0059 |
| L08 vs L11 | **0.0597** | 0.0429 | 0.0757 | **0.0157** | 0.0087 | 0.0269 |
| L08 vs L02 | **0.0747** | 0.0447 | 0.1071 | **0.0184** | 0.012 | 0.0268 |
| L08 vs U03 | **0.0932** | 0.0622 | 0.1235 | **0.0245** | 0.0111 | 0.0363 |
| L08 vs U05 | **0.084** | 0.0396 | 0.1384 | **0.0207** | 0.0127 | 0.041 |
| L08 vs U08 | **0.0568** | 0.0177 | 0.1 | **0.0166** | 0.0085 | 0.0283 |
| L08 vs U10 | **0.0465** | 0.0168 | 0.0892 | **0.0155** | 0.0061 | 0.0303 |
| L08 vs U11 | **0.0878** | 0.0587 | 0.1082 | **0.0212** | 0.0114 | 0.0314 |
| L08 vs U02 | **0.0756** | 0.0499 | 0.0986 | **0.0223** | 0.01 | 0.0342 |
| L08 vs U02 | **0.1077** | 0.0634 | 0.1406 | **0.0407** | 0.0212 | 0.0597 |
| L08 vs V05 | **0.0559** | 0.03 | 0.106 | **0.016** | 0.0085 | 0.0277 |
| L08 vs V08 | 0.0022 | -0.0086 | 0.0271 | 0.0003 | -0.0037 | 0.0083 |
| L08 vs V10 | **0.0293** | 0.0109 | 0.0537 | **0.0082** | 0.0002 | 0.0203 |
| L08 vs V11 | **0.0705** | 0.0525 | 0.0965 | **0.0157** | 0.0102 | 0.0212 |
| L08 vs V02 | **0.0879** | 0.0612 | 0.1328 | **0.0206** | 0.0124 | 0.0323 |
| L10 vs L11 | **0.0175** | 0.0004 | 0.035 | 0.0026 | -0.0016 | 0.0095 |
| L10 vs L02 | **0.0321** | 0.0081 | 0.07 | **0.0052** | 0.0009 | 0.0121 |
| L10 vs U03 | **0.0531** | 0.012 | 0.0847 | **0.0116** | 0.0021 | 0.0194 |
| L10 vs U05 | 0.0393 | -0.0041 | 0.0962 | 0.007 | -0.0018 | 0.0151 |
| L10 vs U08 | 0.0181 | -0.0097 | 0.0501 | 0.0035 | -0.0018 | 0.0075 |
| L10 vs U10 | 0.0098 | -0.01 | 0.0346 | 0.0017 | -0.0019 | 0.0071 |
| L10 vs U11 | **0.0447** | 0.0175 | 0.0775 | **0.0074** | 0.0033 | 0.0127 |
| L10 vs U02 | 0.0256 | -0.0014 | 0.052 | 0.0052 | -0.0014 | 0.0136 |
| L10 vs U02 | **0.0604** | 0.0316 | 0.0822 | **0.0154** | 0.0051 | 0.0231 |
| L10 vs V05 | **0.0289** | 0.0054 | 0.0541 | **0.0051** | 0.0012 | 0.0085 |
| L10 vs V08 | 0.0164 | -0.0065 | 0.0383 | 0.0033 | -0.0033 | 0.0106 |
| L10 vs V10 | 0.0167 | -0.0015 | 0.0322 | 0.0031 | 0 | 0.0078 |
| L10 vs V11 | **0.0547** | 0.0421 | 0.071 | **0.0091** | 0.0077 | 0.0105 |
| L10 vs V02 | **0.0591** | 0.0307 | 0.0901 | **0.0104** | 0.0057 | 0.0153 |
| L11 vs L02 | 0.0001 | -0.0159 | 0.0139 | 0.0001 | -0.0023 | 0.0022 |
| L11 vs U03 | 0.013 | -0.0051 | 0.036 | 0.0016 | -0.0022 | 0.0064 |
| L11 vs U05 | 0.0036 | -0.01 | 0.0344 | 0 | -0.0033 | 0.0111 |
| L11 vs U08 | -0.0006 | -0.0319 | 0.0428 | 0 | -0.0049 | 0.0061 |
| L11 vs U10 | 0.0033 | -0.0122 | 0.018 | 0.0005 | -0.0021 | 0.0033 |
| L11 vs U11 | 0.0066 | -0.0108 | 0.0401 | 0.0007 | -0.0015 | 0.0042 |
| L11 vs U02 | -0.0016 | -0.0148 | 0.0158 | -0.0002 | -0.0025 | 0.0058 |
| L11 vs U02 | **0.0385** | 0.0088 | 0.062 | 0.0065 | -0.0002 | 0.0172 |
| L11 vs V05 | 0.0219 | -0.0021 | 0.0619 | 0.0031 | -0.0003 | 0.01 |
| L11 vs V08 | **0.0663** | 0.027 | 0.0908 | **0.0141** | 0.0037 | 0.0219 |
| L11 vs V10 | **0.0467** | 0.0119 | 0.0802 | **0.0079** | 0.0018 | 0.0148 |
| L11 vs V11 | **0.0582** | 0.0369 | 0.0755 | **0.0082** | 0.0062 | 0.0108 |
| L11 vs V02 | **0.0478** | 0.0205 | 0.0724 | **0.0068** | 0.0039 | 0.0102 |
| L02 vs U03 | **0.0213** | 0.0028 | 0.0448 | 0.0012 | -0.0029 | 0.0062 |
| L02 vs U05 | **0.0096** | 0.001 | 0.0146 | 0.0007 | -0.002 | 0.0034 |
| L02 vs U08 | 0.0046 | -0.0143 | 0.0336 | 0.0006 | -0.0037 | 0.009 |
| L02 vs U10 | 0.003 | -0.0071 | 0.0171 | 0.0005 | -0.002 | 0.0035 |
| L02 vs U11 | -0.0016 | -0.0164 | 0.0156 | -0.0001 | -0.0025 | 0.0029 |
| L02 vs U02 | 0.0024 | -0.0098 | 0.0185 | 0.0002 | -0.0023 | 0.0039 |
| L02 vs U02 | **0.0376** | 0.0109 | 0.0582 | 0.006 | -0.0003 | 0.0127 |
| L02 vs V05 | 0.013 | -0.0092 | 0.044 | 0.0017 | -0.0027 | 0.0078 |
| L02 vs V08 | **0.0793** | 0.0463 | 0.1035 | **0.0176** | 0.0092 | 0.0248 |
| L02 vs V10 | **0.0518** | 0.0165 | 0.0805 | **0.0093** | 0.0021 | 0.0175 |
| L02 vs V11 | **0.0704** | 0.0429 | 0.1003 | **0.0108** | 0.0067 | 0.0151 |
| L02 vs V02 | **0.0432** | 0.0203 | 0.0606 | **0.0065** | 0.0027 | 0.0108 |
| U03 vs U05 | 0.0132 | -0.0017 | 0.0459 | 0.0008 | -0.0027 | 0.0095 |
| U03 vs U08 | 0.0011 | -0.0152 | 0.0211 | 0 | -0.0029 | 0.0056 |
| U03 vs U10 | **0.0193** | 0.0023 | 0.0359 | 0.0039 | -0.001 | 0.0094 |
| U03 vs U11 | 0.0017 | -0.0145 | 0.0272 | 0 | -0.0025 | 0.0043 |
| U03 vs U02 | 0.0149 | -0.0056 | 0.0355 | 0.001 | -0.0041 | 0.009 |
| U03 vs U02 | **0.0471** | 0.0213 | 0.0702 | **0.0079** | 0.0024 | 0.0169 |
| U03 vs V05 | **0.0336** | 0.0111 | 0.0631 | **0.0066** | 0.0017 | 0.0134 |
| U03 vs V08 | **0.0852** | 0.0424 | 0.1303 | **0.0178** | 0.004 | 0.0304 |
| U03 vs V10 | **0.0809** | 0.0476 | 0.1272 | **0.0166** | 0.0104 | 0.0309 |
| U03 vs V11 | **0.0788** | 0.0415 | 0.1129 | **0.0109** | 0.0066 | 0.0144 |
| U03 vs V02 | **0.0579** | 0.0306 | 0.0861 | **0.0079** | 0.0041 | 0.0141 |
| U05 vs U08 | 0.002 | -0.0144 | 0.0204 | 0.0002 | -0.003 | 0.006 |
| U05 vs U10 | 0.0102 | -0.0099 | 0.0344 | 0.0014 | -0.0021 | 0.0057 |
| U05 vs U11 | 0.0099 | -0.0061 | 0.0285 | 0.0008 | -0.0021 | 0.0044 |
| U05 vs U02 | 0.0044 | -0.011 | 0.0233 | 0 | -0.0023 | 0.0039 |
| U05 vs U02 | **0.0375** | 0.0242 | 0.0617 | **0.0058** | 0.003 | 0.0129 |
| U05 vs V05 | **0.0353** | 0.0068 | 0.0593 | **0.0063** | 0.0009 | 0.0108 |
| U05 vs V08 | **0.0778** | 0.0214 | 0.1289 | **0.0159** | 0.0006 | 0.0366 |
| U05 vs V10 | **0.0664** | 0.0483 | 0.0939 | **0.0124** | 0.009 | 0.0171 |
| U05 vs V11 | **0.0734** | 0.0315 | 0.1065 | **0.011** | 0.0053 | 0.0169 |
| U05 vs V02 | **0.0503** | 0.0222 | 0.08 | **0.0073** | 0.0029 | 0.0113 |
| U08 vs U10 | -0.002 | -0.017 | 0.0312 | -0.0004 | -0.0035 | 0.0061 |
| U08 vs U11 | -0.0001 | -0.0225 | 0.0271 | 0 | -0.0037 | 0.0042 |
| U08 vs U02 | 0.0037 | -0.0207 | 0.0494 | 0 | -0.0055 | 0.0087 |
| U08 vs U02 | 0.0182 | -0.0019 | 0.0537 | 0.0038 | -0.0013 | 0.012 |
| U08 vs V05 | 0.0059 | -0.0046 | 0.0141 | 0.0011 | -0.0011 | 0.0026 |
| U08 vs V08 | **0.0452** | 0.0297 | 0.0663 | **0.011** | 0.0035 | 0.0227 |
| U08 vs V10 | **0.035** | 0.0107 | 0.0649 | **0.0075** | 0.0011 | 0.0157 |
| U08 vs V11 | **0.0401** | 0.0136 | 0.0799 | **0.0065** | 0.0029 | 0.0097 |
| U08 vs V02 | **0.025** | 0.0099 | 0.039 | **0.0041** | 0.0019 | 0.0056 |
| U10 vs U11 | 0.0052 | -0.0085 | 0.0191 | 0.0006 | -0.0015 | 0.0028 |
| U10 vs U02 | 0.004 | -0.0132 | 0.0181 | 0.0004 | -0.0027 | 0.0052 |
| U10 vs U02 | 0.0145 | -0.0049 | 0.0302 | 0.0026 | -0.0031 | 0.0099 |
| U10 vs V05 | 0.0021 | -0.0101 | 0.0151 | 0.0003 | -0.002 | 0.0033 |
| U10 vs V08 | **0.04** | 0.0053 | 0.0632 | 0.0115 | -0.0008 | 0.0276 |
| U10 vs V10 | 0.0208 | -0.003 | 0.0537 | 0.0048 | -0.0017 | 0.0147 |
| U10 vs V11 | **0.0356** | 0.0173 | 0.0625 | **0.0064** | 0.0037 | 0.01 |
| U10 vs V02 | **0.0217** | 0.0054 | 0.0448 | **0.0038** | 0.0013 | 0.0081 |
| U11 vs U02 | 0.0017 | -0.0184 | 0.0197 | 0.0001 | -0.0028 | 0.0029 |
| U11 vs U02 | **0.0309** | 0.0167 | 0.0608 | **0.0046** | 0.002 | 0.012 |
| U11 vs V05 | **0.0145** | 0.0009 | 0.0362 | 0.0025 | -0.0003 | 0.0066 |
| U11 vs V08 | **0.0831** | 0.0453 | 0.1126 | **0.0177** | 0.0055 | 0.0327 |
| U11 vs V10 | **0.0601** | 0.0268 | 0.0925 | **0.0115** | 0.007 | 0.0173 |
| U11 vs V11 | **0.0642** | 0.0452 | 0.0816 | **0.009** | 0.0059 | 0.011 |
| U11 vs V02 | **0.0359** | 0.0119 | 0.0716 | **0.0047** | 0.0015 | 0.0079 |
| U02 vs U02 | **0.0317** | 0.0036 | 0.0546 | 0.0049 | -0.0011 | 0.0147 |
| U02 vs V05 | **0.0351** | 0.0111 | 0.0583 | **0.0066** | 0.002 | 0.0123 |
| U02 vs V08 | **0.0787** | 0.0405 | 0.105 | **0.0188** | 0.0052 | 0.0344 |
| U02 vs V10 | **0.0572** | 0.0354 | 0.0896 | **0.0118** | 0.0065 | 0.0199 |
| U02 vs V11 | **0.0673** | 0.0356 | 0.0996 | **0.0107** | 0.0056 | 0.0182 |
| U02 vs V02 | **0.0386** | 0.0178 | 0.0605 | **0.0057** | 0.0028 | 0.0088 |
| U02 vs V05 | **0.0274** | 0.0037 | 0.0529 | 0.0065 | -0.0001 | 0.014 |
| U02 vs V08 | **0.0745** | 0.021 | 0.1067 | **0.0317** | 0.0049 | 0.0502 |
| U02 vs V10 | **0.0437** | 0.012 | 0.0842 | **0.0173** | 0.0019 | 0.0368 |
| U02 vs V11 | 0.0244 | -0.0017 | 0.0521 | 0.004 | -0.0039 | 0.0134 |
| U02 vs V02 | 0.0041 | -0.0102 | 0.0181 | 0.0001 | -0.0029 | 0.0041 |
| V05 vs V08 | **0.0434** | 0.0005 | 0.0767 | 0.0121 | -0.0007 | 0.0228 |
| V05 vs V10 | **0.0221** | 0.0001 | 0.0387 | 0.0048 | -0.0009 | 0.0106 |
| V05 vs V11 | **0.0333** | 0.0081 | 0.062 | **0.0056** | 0.002 | 0.0101 |
| V05 vs V02 | **0.0269** | 0.0096 | 0.0545 | **0.0046** | 0.0014 | 0.0096 |
| V08 vs V10 | 0.0148 | -0.0008 | 0.0333 | 0.0013 | -0.0035 | 0.0119 |
| V08 vs V11 | **0.0422** | 0.0151 | 0.0767 | **0.0108** | 0.0017 | 0.0215 |
| V08 vs V02 | **0.0543** | 0.0077 | 0.0826 | **0.0134** | 0.0003 | 0.0204 |
| V10 vs V11 | **0.0181** | 0.0047 | 0.0414 | **0.0049** | 0.0013 | 0.0136 |
| V10 vs V02 | **0.0317** | 0.0113 | 0.0635 | **0.0092** | 0.0033 | 0.018 |
| V11 vs V02 | 0.0053 | -0.0119 | 0.038 | 0.0006 | -0.0028 | 0.005 |

Supplementary Table S2: Genetic differentiation between cohorts within sites estimated via G´_ST_ and the respective confidence intervals (CI). Values are significant when 0 is not included in the CI and are displayed in bold.

|  |  | G´_ST_ | | |
| --- | --- | --- | --- | --- |
|  | comparison | actual | lower CI | upper CI |
| Lynæs | cohort1 - cohort2 | 0.012 | -0.0038 | 0.0217 |
|  | cohort1- cohort3 | **0.0243** | **0.0016** | **0.0818** |
|  | cohort1-cohort4 | 0.0148 | -0.0007 | 0.0321 |
|  | cohort2-cohort3 | **0.0193** | **0.0038** | **0.0446** |
|  | cohort2-cohort4 | 0.0065 | -0.0131 | 0.0221 |
|  | cohort3-cohort4 | **0.0124** | **0.001** | **0.0281** |
| Lammefjord | cohort1 - cohort2 | **0.0826** | **0.0609** | **0.1242** |
|  | cohort1- cohort3 | 0.0113 | -0.0025 | 0.0349 |
|  | cohort2-cohort3 | **0.0694** | **0.0229** | **0.1263** |
| Herslev | cohort1 - cohort2 | 0.0099 | -0.0082 | 0.0426 |
|  | cohort1- cohort3 | 0.0063 | -0.0012 | 0.0137 |
|  | cohort2-cohort3 | **0.0138** | **0.0003** | **0.0261** |

Supplementary Table S3: Genetic differentiation between females and males respectively within site estimated via G´_ST_ and the respective confidence intervals (CI). Values are significant when 0 is not included in the CI and are displayed in bold. No statistics can be calculated when less than two individuals are in one sample or when there are less than three samples per comparison. Hence, no results are available for Lynæs, for Lammefjord females and males in May and August, for Vellerup females in May and August and for Herslev males in August.

|  |  |  | G´_ST_ | | |
| --- | --- | --- | --- | --- | --- |
|  |  | comparison | actual | lower CI | upper CI |
| Lammefjord | Females | Mar - Oct | **0.213** | **0.155** | **0.299** |
|  |  | Mar - Nov | **0.207** | **0.140** | **0.281** |
|  |  | Mar - Feb | **0.123** | **0.042** | **0.230** |
|  |  | Oct - Nov | -0.001 | -0.112 | 0.144 |
|  |  | Oct - Feb | **0.114** | **0.059** | **0.181** |
|  |  | Nov - Feb | 0.002 | -0.094 | 0.114 |
|  | Males | Mar - Oct | **0.298** | **0.198** | **0.508** |
|  |  | Mar - Nov | **0.169** | **0.038** | **0.301** |
|  |  | Mar - Feb | -0.027 | -0.134 | 0.103 |
|  |  | Oct - Nov | -0.033 | -0.132 | 0.143 |
|  |  | Oct - Feb | **0.180** | **0.042** | **0.403** |
|  |  | Nov - Feb | 0.051 | -0.081 | 0.246 |
| Vellerup | Females | Mar - Oct | **0.211** | **0.132** | **0.324** |
|  |  | Mar - Nov | **0.115** | **0.053** | **0.169** |
|  |  | Mar - Feb | -0.014 | -0.082 | 0.067 |
|  |  | Oct - Nov | 0.014 | -0.060 | 0.094 |
|  |  | Oct - Feb | 0.105 | -0.035 | 0.300 |
|  |  | Nov - Feb | 0.031 | -0.058 | 0.174 |
|  | Males | Mar - May | **0.178** | **0.094** | **0.282** |
|  |  | Mar - Aug | **0.146** | **0.113** | **0.202** |
|  |  | Mar - Oct | **0.127** | **0.054** | **0.228** |
|  |  | Mar - Nov | **0.068** | **0.034** | **0.105** |
|  |  | Mar - Feb | **0.025** | **0.006** | **0.084** |
|  |  | May - Aug | **0.517** | **0.411** | **0.598** |
|  |  | May - Oct | **0.182** | **0.045** | **0.307** |
|  |  | May - Nov | **0.273** | **0.126** | **0.412** |
|  |  | May - Feb | **0.164** | **0.056** | **0.296** |
|  |  | Aug - Oct | **0.186** | **0.066** | **0.346** |
|  |  | Aug - Nov | -0.038 | -0.120 | 0.089 |
|  |  | Aug - Feb | -0.005 | -0.030 | 0.024 |
|  |  | Oct - Nov | 0.106 | -0.016 | 0.252 |
|  |  | Oct - Feb | 0.060 | -0.055 | 0.197 |
|  |  | Nov - Feb | -0.021 | -0.069 | 0.035 |
| Herslev | Females | Mar - May | -0.007 | -0.079 | 0.105 |
|  |  | Mar - Aug | 0.044 | -0.078 | 0.199 |
|  |  | Mar - Oct | 0.018 | -0.128 | 0.186 |
|  |  | Mar - Nov | 0.005 | -0.025 | 0.064 |
|  |  | Mar - Feb | 0.011 | -0.037 | 0.066 |
|  |  | May - Aug | -0.034 | -0.160 | 0.121 |
|  |  | May - Oct | 0.081 | -0.070 | 0.332 |
|  |  | May - Nov | -0.019 | -0.103 | 0.114 |
|  |  | May - Feb | -0.011 | -0.056 | 0.102 |
|  |  | Aug - Oct | 0.014 | -0.173 | 0.219 |
|  |  | Aug - Nov | 0.053 | -0.068 | 0.204 |
|  |  | Aug - Feb | 0.068 | -0.048 | 0.212 |
|  |  | Oct - Nov | 0.133 | -0.020 | 0.361 |
|  |  | Oct - Feb | 0.044 | -0.046 | 0.167 |
|  |  | Nov - Feb | 0.031 | -0.015 | 0.105 |
|  | Males | Mar - May | -0.012 | -0.055 | 0.035 |
|  |  | Mar - Oct | -0.008 | -0.064 | 0.123 |
|  |  | Mar - Nov | -0.020 | -0.036 | 0.013 |
|  |  | Mar - Feb | -0.008 | -0.018 | 0.010 |
|  |  | May - Oct | 0.037 | -0.051 | 0.174 |
|  |  | May - Nov | -0.024 | -0.076 | 0.019 |
|  |  | May - Feb | -0.011 | -0.098 | 0.071 |
|  |  | Oct - Nov | 0.004 | -0.066 | 0.137 |
|  |  | Oct - Feb | 0.009 | -0.057 | 0.084 |
|  |  | Nov - Feb | -0.004 | -0.029 | 0.019 |

Supplementary Table S4: Genetic differentiation between females, males and all specimens within samples estimated via G´_ST_ and the respective confidence intervals (CI). Values are significant when positive and when 0 is not included in the CI and are displayed in bold. No statistics can be calculated when less than two individuals are in one sample or when there are less than three samples per comparison. Hence, no results are available for Lynæs except in October, for Lammefjord and Vellerup in May and August and for Herslev in August.

|  |  |  | G´_ST_ | | |
| --- | --- | --- | --- | --- | --- |
|  |  | comparison | actual | lower CI | upper CI |
| Lynæs | Oct | females - males | 0.168 | -0.017 | 0.319 |
|  |  | females - total | **0.100** | **0.010** | **0.245** |
|  |  | males - total | **0.167** | **0.086** | **0.274** |
| Lammefjord | Mar | females - males | 0.013 | -0.020 | 0.045 |
|  |  | females - total | -0.016 | -0.024 | -0.007 |
|  |  | males - total | 0.007 | -0.034 | 0.069 |
|  | Oct | females - males | 0.035 | -0.028 | 0.137 |
|  |  | females - total | **0.050** | **0.012** | **0.113** |
|  |  | males - total | **0.072** | **0.017** | **0.161** |
|  | Nov | females - males | -0.005 | -0.189 | 0.238 |
|  |  | females - total | **0.156** | **0.023** | **0.341** |
|  |  | males - total | **0.061** | **0.011** | **0.115** |
|  | Feb | females - males | 0.088 | -0.042 | 0.279 |
|  |  | females - total | 0.085 | -0.054 | 0.258 |
|  |  | males - total | -0.030 | -0.098 | 0.096 |
| Vellerup | Mar | females - males | -0.006 | -0.040 | 0.026 |
|  |  | females - total | -0.018 | -0.034 | 0.009 |
|  |  | males - total | -0.016 | -0.038 | 0.016 |
|  | Oct | females - males | 0.002 | -0.101 | 0.166 |
|  |  | females - total | 0.032 | -0.027 | 0.100 |
|  |  | males - total | 0.006 | -0.067 | 0.101 |
|  | Nov | females - males | 0.062 | -0.018 | 0.174 |
|  |  | females - total | 0.028 | -0.006 | 0.124 |
|  |  | males - total | -0.004 | -0.028 | 0.023 |
|  | Feb | females - males | -0.064 | -0.141 | 0.101 |
|  |  | females - total | -0.019 | -0.062 | 0.038 |
|  |  | males - total | -0.009 | -0.039 | 0.055 |
| Herslev | Mar | females - males | -0.016 | -0.033 | 0.002 |
|  |  | females - total | -0.013 | -0.026 | 0.001 |
|  |  | males - total | -0.019 | -0.032 | -0.009 |
|  | May | females - males | -0.055 | -0.142 | 0.041 |
|  |  | females - total | -0.025 | -0.088 | 0.026 |
|  |  | males - total | -0.022 | -0.064 | 0.044 |
|  | Oct | females - males | -0.088 | -0.195 | 0.052 |
|  |  | females - total | -0.053 | -0.112 | 0.111 |
|  |  | males - total | -0.021 | -0.072 | 0.051 |
|  | Nov | females - males | -0.019 | -0.064 | 0.036 |
|  |  | females - total | 0.003 | -0.035 | 0.057 |
|  |  | males - total | -0.012 | -0.030 | 0.012 |
|  | Feb | females - males | -0.019 | -0.053 | 0.023 |
|  |  | females - total | -0.016 | -0.055 | 0.109 |
|  |  | males - total | -0.008 | -0.036 | 0.026 |


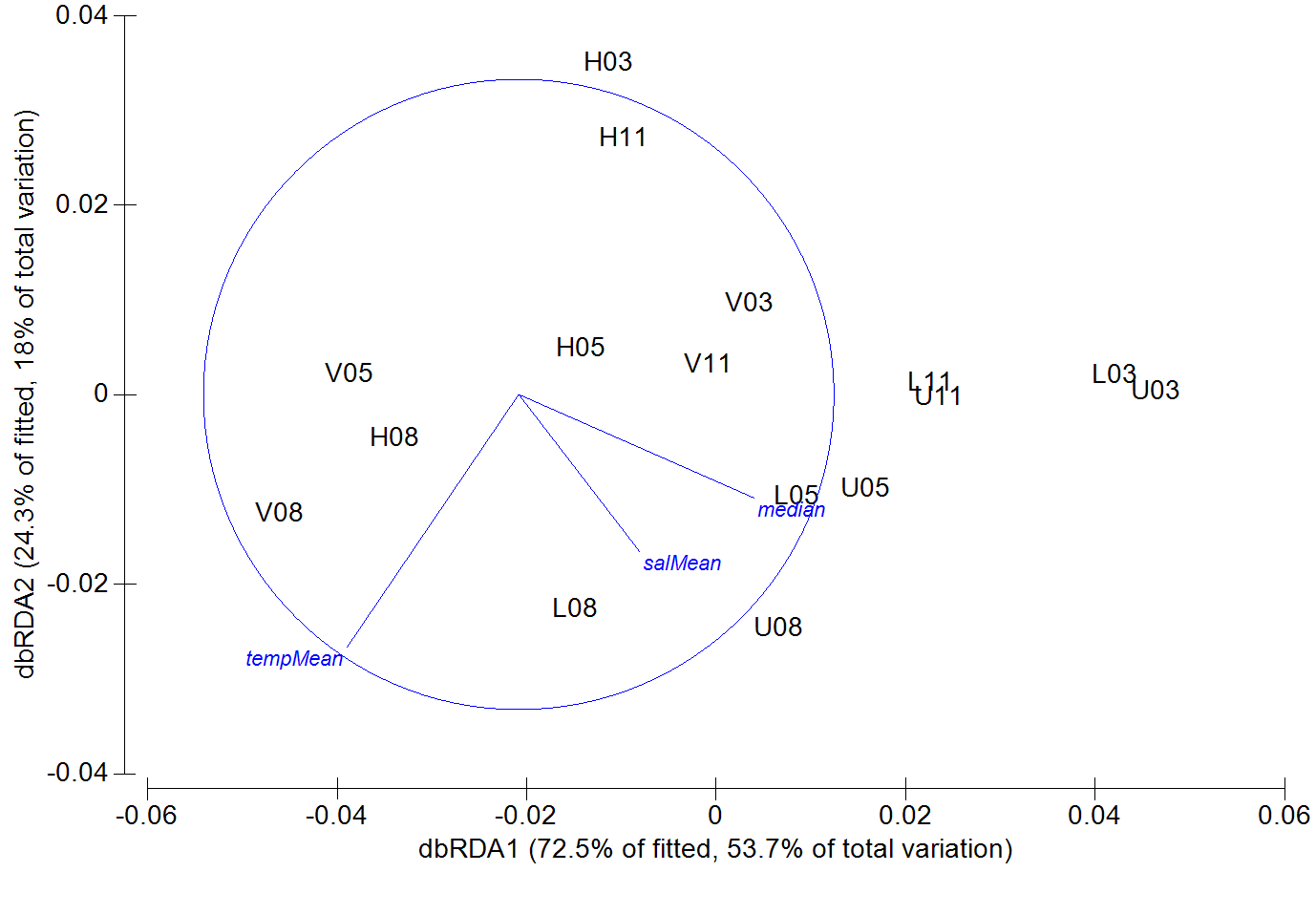


Supplementary Figure S1: Distance-based redundancy analysis (dbRDA): Ordination of the population genetic differences of *Pygospio elegans* between the four sites and time points(U, Lynæs; L, Lammefjord; V, Vellerup; H, Herslev; 3, March; 5, May; 8, August; 11, November) fitted to the significant predictor environmental parameters temperature, median grain size, and salinity. The parameters explain 74% of the total variation in the population genetic structure, with 72% explained by the first two axes as shown. Overlaid vectors indicate the loadings (importance) of the predictor parameters temperature, median grain size, and salinity on the two axes.
